# Characterization of active Cerish fructan‐sumac extract composite films: Physical, mechanical, and antioxidant properties

**DOI:** 10.1002/fsn3.3406

**Published:** 2023-05-04

**Authors:** Somaye Pakseresht, Jouhaina Hadree, Nasser Sedaghat

**Affiliations:** ^1^ Department of Food Science and Technology, Faculty of agriculture Ferdowsi University of Mashhad (FUM) Mashhad Iran

**Keywords:** DPPH, DSC, *Eremurus spectabilis*, reducing power activity, *Rhus coriaria* L., XRD

## Abstract

The biodegradable active films have the potential to increase the shelf life and safety of food products. In this study, the properties of *Eremurus spectabilis* (Cerish) root fructans (ESRF) film and its combination with *Rhus coriaria* L. (Sumac) extract (RCLE) at different concentrations (1%, 2%, 3%, and 4% w/w) were investigated. The Fourier transform infrared (FTIR) analysis determined the fingerprint region of fructans at 950–1150 cm^−1^ in all spectrograms. RCLE increased the interactions between the hydroxyl groups and the formation of intermolecular bonds in composite films. Elongation‐at‐break (EAB) and tensile strength (TS) did not change significantly. However, RCLE increased Young's modulus (YM) (*p* ˂ .05), thermal stability, and crystallinity of composite films. RCLE also increased the film thickness and decreased the water content, solubility, and swelling degree significantly. RCLE improved the reducing ability and free radical‐scavenging activity of composite films. Present results indicated that the ESRF/RCLE films were the protective barriers to the permeability of water vapor. The incorporation of RCLE increased the surface hydrophobicity and caused the composite film microstructure to become uniform and more compact. Overall, the Sumac extract at the specific concentration of 3% can be used to improve the Cerish fructans film properties and extend the product's shelf life in active food packaging.

## Graphical abstract

In this study, the properties of *Eremurus spectabilis* (Cerish) root fructans (ESRF) film and its combination with *Rhus coriaria* L. (Sumac) extract (RCLE) at different concentrations (1%, 2%, 3%, and 4 % w/w) were investigated. The Sumac extract at the specific concentration of 3% can be used to improve the Cerish fructans film properties and extend the product's shelf life in active food packaging.

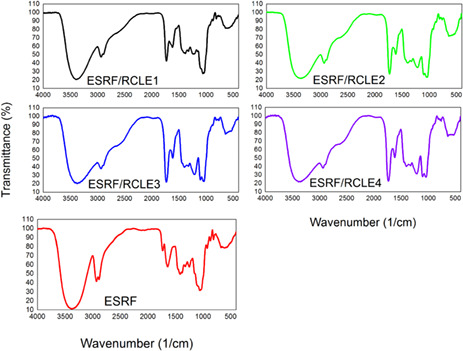

## 1 INTRODUCTION

Synthetic food packaging films, which are produced from petroleum‐based polymers, are a source of air and soil pollution. In contrast, biodegradable packaging materials, which are based on proteins, polysaccharides, and lipids, have a less detrimental effect on the environment (Mozafarpour et al., 2021; Yang et al., 2016). These biodegradable films/coatings have the potential to reduce the plastic usage. This replacement decreases the food packaging waste streams. The packaging materials should be food grade and maintain/increase the shelf‐life stability and safety of the product (Wang et al., 2007).

In recent years, polysaccharide‐based biofilms have gained considerable interest because they are renewable, biodegradable, nontoxic, and economical biopolymers. Furthermore, the gas barrier characteristics of polysaccharide‐based films are good. Therefore, the respiration and ripening processes of vegetables and fruits are delayed (Mozafarpour et al., 2021). Cellulose and its derivatives (carboxymethyl cellulose (CMC), methylcellulose (MC), and hydroxypropyl methylcellulose (HPMC)), xanthan gum, psyllium seed gum, tapioca, corn, potato, and rice starch are typical examples of available and economic polysaccharides with the film‐forming potential (Jouki et al., 2013; Wang et al., 2007). However, the production of biodegradable and edible films from new sources, such as cress seed carbohydrate gum (Jouki et al., 2013), gum ghatti (Zhang et al., 2016), tara gum (Antoniou et al., 2014), and sage seed gum (Razavi et al., 2015) has gained attention over the past few years.

*Eremurus spectabilis* (also known as Cirish or Cerish) belongs to the *Eremurus* (Liliaceae) genus, which is widely distributed in the Middle East and central and western Asia. Cerish roots, as a folk remedy, have conventionally been used to cure several diseases, including jaundice, stomach irritation, liver disorders, bone fractures, and pimples. It has also been used as natural glue in the industry (Pourfarzad et al., 2015). The phenolic compounds of *E*. *spectabilis* showed an antiproliferative effect against prostate cancer cells (Tuzcu et al., 2017). The major chemical composition of *Eremurus* roots is fructans. Fructans are natural oligo‐ and polysaccharides consisting of D‐fructose chains and a single D‐glucose unit at the non‐reducing end. Inulin, the main component of fructans, is a polysaccharide, with generally the degree of polymerization from 2 to 60. It has beneficial nutritional properties. In addition to its prebiotic benefit aspect, inulin has technofunctional properties. It has been used in dairy, bakery, and confectionery products to increase the rheological, textural, and sensory characteristics (Pourfarzad et al., 2015; Temkov et al., 2015).

Several studies have been carried out to study the *E*. *spectabilis* properties. Pourfarzad et al. (2015) studied the effect of different drying methods (freeze‐drying, spray drying, vacuum drying, and oven drying) on the physicochemical properties of ESRF. They reported that the drying method did not affect the fructans' structure. However, the changes in the microstructure of fructan particles were significant, causing differences in physicochemical properties. Different extracts (chloroform, toluene, ethyl acetate, and aqueous extract) of Cerish young leaves were examined by Taskin et al. (2012) to evaluate antimicrobial and antioxidant activities. They reported that the aqueous extract showed protection against *Candida albicans* and *Staphylococcus aureus*, while the ethyl acetate extract had the highest antioxidant activity. They recommended the consumption of young leaves as a natural antioxidant source. Tosun et al. (2012) introduced the young leaves of *E*. *spectabilis* as a rich source of minerals (K, P, Ca, and Mg), phenolic, and antioxidant components. Tuzcu et al. (2017) also compared the organic (acetone and ethanol extracts) and water extracts of both Cerish roots and leaves. They reported that the acetone extract of leaves had the highest antioxidant activity.

Antioxidant compounds can delay the oxidation process by scavenging free radicals, chelating metals, or giving hydrogen atoms. However, the legislations restrict the content of synthetic antioxidants usage because of uncertainties about their toxicity and carcinogenicity. Therefore, consumers have a preference for natural antioxidants. Grains, herbs, spices, fruits, and vegetables are natural sources of antioxidants (Bursal & Köksal, 2011; Kosar et al., 2007).

*Rhus coriaria* L. (also known as Sumac) belongs to the Anacardiaceae family, which is a rich source of anthocyanins (cyanidin, peonidin, pelargonidin, petunidin, delphinidin glucosides, and coumarates), hydrolyzable tannins, and gallic acid with the antioxidant activity (Kosar et al., 2007). Compared with the ethanol extracts, the water extracts of *Rhus coriaria* L. showed higher antioxidant and radical‐scavenging activities (Bursal & Köksal, 2011).

Regarding the rheological, chemical, and functional properties of *E*. *spectabilis*, it seems that ESR fructans are a suitable raw material for the preparation of biodegradable films. Therefore, the objective of the present study was the production of a new biodegradable film using Cerish root polysaccharides. However, owing to the higher antioxidant capacity of its leaves, we try to reinforce its antioxidant properties by RCLE.

## 2 MATERIALS AND METHODS

The *E*. *spectabilis* root powder and *Rhus coriaria* L. powder were purchased from the local medicinal plant market. The *E*. *spectabilis* root powder was stored in a dry container after sifting by a 50 μm sieve for further use. Glycerol was purchased from Sigma‐Aldrich Pte Ltd. Other chemicals were purchased from Merck‐Germany.

### 2.1 Extraction of fructans

To extract aqueous fructans and facilitate its solubility, *E*. *spectabilis* root powder was suspended in distilled water at a ratio of 1:50 (w/v) and heated at 85–90°C for 30 min in the water bath (Memmert Company, model WB/0B7‐45). The insoluble residues were separated by muslin cloth. Then the supernatant was collected by centrifuging (Sigma, 4‐16KS model, Osterede, Germany) the slurry at 4500 *g* for 10 min. Finally, the fructans extract was dried at 50°C for 24–48 h using a hot air oven (Memmert Company) and packaged in the air‐tight‐container before the film preparation (Pourfarzad et al., 2015, with some modification).

### 2.2 Water extract of *Rhus coriaria* L.

The water extraction method of *Rhus coriaria* L. was performed according to Bursal and Köksal (2011) with some modifications. Briefly, *Rhus coriaria* L. powder was added to the distilled water at a ratio of 1:10 (w/v) and heated at 100°C for 10 min with magnetic stirring. Then the extract was filtered using a muslin cloth, centrifuged (6000 rpm, 10 min), and filtered with filter paper, respectively. The resulting extract was dried using a hot air oven (40°C, 48–72 h) and packed in the air‐tight container.

### 2.3 Film preparation

A 2% fructans extract solution, according to the preliminary tests, was prepared by the addition of the extracted powder in distilled water. The solution was shaken for 24 h using the magnetic stirrer and then heated at 80°C for 30 min. Glycerol, as a plasticizer, was added to the hot solution at 60% w/w (based on the dry weight) and stored for 15 min. Afterward, the solution was cooled to room temperature (25°C), and *Rhus coriaria* L. extracted powder was added at 1%, 2%, 3%, and 4% w/w. The solutions were magnetically stirred for 60 min. The films were formed by casting the resulting solutions onto Petri dishes and drying them in a hot air oven for 24 h at 40°C. Then, the films were peeled off and put in a desiccator before further testing. The sample without the sumac extracted powder was used as a control.

### 2.4 Fourier transform infrared (FTIR) spectroscopy

The structural interactions between the ESRF and RCLE were investigated by the FTIR spectrometer (Thermo Nicolet, AVATRA 370 FT‐IR). The samples were scanned in spectra ranging from 400 to 4000 cm^−1^.

### 2.5 Mechanical properties

The mechanical properties of films, including tensile strength, elongation‐at‐break (EAB), and Young's modulus, were measured by the texture analyzer (TA‐XT Plus TM, Stable Micro Systems, England). At first, films were cut into strips (100 × 6 mm). Then, the strip ends were mounted between metal grips with an initial separation of 50 mm. The crosshead speed was 60 mm/min. Five replication was done for each sample. Tensile strength (MPa) was calculated according to the following equation:

$$\text{Tensile strength}=\frac{F}{x\times W}.$$

Where *F* is the maximum stretching force (*N*), *x* and *W* are the thickness and width of film (mm) respectively.

The following equation was used to calculate EAB:

$$\mathrm{EAB}\;\left(\%\right)=\frac{L_{1}-L_{0}}{L_{0}}\times 100$$

where *L*_1_ is the fracture length (mm), *L*_0_ is the original length of film (mm) (Liu et al., 2017).

### 2.6 Glass transition temperature (Tg)

The glass transition temperature of samples was investigated by differential scanning calorimetry (DSC) (Malvern, Nano DSC, Zen3600). Approximately, 18 mg of each film was sealed in an aluminum pan and heated from room temperature (25°C) to 250°C with a heating rate of 10°C min^−1^. As all curves exhibited a broad peak, the onset temperature of the peak was considered as the glass transition temperature (Tg). The examination was carried out in triplicate.

### 2.7 Physical properties

#### 2.7.1 Film thickness

A digital micrometer device (QLR digit‐IP54, China) was used to evaluate the thickness of the prepared films. The thickness of 10 different random points was measured. The examination was carried out in triplicate (de Moraes Crizel et al., 2018).

#### 2.7.2 Water content, solubility and swelling degree

Films were cut into a rectangle 2 × 2 cm and were weighted (M_1_). Then the samples were dried using a hot air oven (70°C, 24 h). The dried mass was then determined (M_2_). Subsequently, the film specimens were immersed in 30 mL distilled water (25°C). After overnight, samples were removed, wiped with filter paper, and weighted (M_3_). The residual films were dried (70°C, 24 h), and their final dry mass was determined (M_4_). Water content, solubility, and swelling degree were calculated by the following equations (Souza et al., 2017):

$$\text{Water content}\;\left(\%\right)=\frac{M_{1}-M_{2}}{M_{1}}\times 100$$

$$\text{Solubility}\;\left(\%\right)=\frac{M_{2}-M_{4}}{M_{2}}\times 100$$

$$\text{Swelling degree}\;\left(\%\right)=\frac{M_{3}-M_{2}}{M_{2}}\times 100$$

### 2.8 Antioxidant activity

#### 2.8.1 DPPH free Radical‐Scavenging activity

The ability of films to act as a free radical scavenger was evaluated according to Liu et al. (2017) with some modification. At first, the film samples were dissolved in methanol (0.4 mg/mL). Then 0.5 mL of each solution was added to 3 mL methanol and 1.5 mL DPPH (0.2 mM) and kept in a dark place for 45 min at room temperature. Finally, the absorbance of the control (*A*_0_) and film sample containing solutions (*A*_1_) was measured at *λ* = 517 nm. The following equation was used to calculate the DPPH free radical‐scavenging activity:

$$\text{DPPH free radical}-\text{scavenging activity}\;\left(\%\right)=\frac{A_{0}-A_{1}}{A_{0}}\times 100$$

#### 2.8.2 Reducing power activity

The iron (III) reduction ability of films was measured according to Benzie and Strain (1996) with some modification. In brief, 10 mL acetate buffer (0.3 M, pH 3.6), 1 mL TPTZ (2,4, 6‐tripyridyl‐S‐triazine) solution (23.4 mg of TPTZ in 7.5 mL of 40 mM HCl), and 1 mL ferric solution (FeCl_3_ 6 H_2_O) (20 mM) were mixed to prepare the FRAP reagent. Then 0.075 mL films' methanolic solution (0.04 mg/mL) was mixed with 2.25 mL FRAP working reagent and 0.225 mL distilled water, kept in a dark place for 10 min at 37°C, and taken the absorbance at *λ* = 593 nm against the blank solution. FeSO_4_. 7H_2_O (200–2000 μmoL/L) was used to prepare the standard curve. The results were expressed in μM. All solutions were freshly prepared.

### 2.9 Water vapor permeability (WVP)

The rate of water vapor transmission of the films was measured according to ASTM (1995), with some modifications. In this method, the moisture absorption material was anhydrous calcium chloride (RH = 0%). Vials (2 cm diameter and 5 cm height) with an air gap equal to 0.5 cm at their upper bungs were used. They contained 4 g calcium chloride. Films were cut and placed between the mouth and bungs of the vials. The relative humidity and temperature of the vials' chamber were 95% ± 3% and 25 ± 1°C, respectively. The transmission rate was determined by plotting the weight variation against time. The water vapor transmission (WVT) and water vapor permeability (WVP) were calculated by the following equation:

$$\mathrm{WVT}=\frac{G}{t\times A}$$

$$\text{Permeance}=\frac{\mathrm{WVT}}{S\times \left(R1-R2\right)}$$

$$\mathrm{WVP}\;\text{or average permeability}=\text{Permeance}\times \text{thickness}=\frac{G\times X}{t\times A\times S\times \left(R1-R2\right)}$$

Where *G* is the weight change (*g*), t is the time during which *G* occurred (s), *G*/*t* is the slope of the straight line, *X* is the film thickness (*m*), A is the test area (m^2^), S is the saturated vapor pressure at test temperature (Pa), *R*_1_ is the relative humidity of the measuring medium, and *R*_2_ is the relative humidity of the vials inside.

### 2.10 Contact angle

The contact angles of the film samples were evaluated using a sessile drop technique. Briefly, a drop (5 μL) of distilled water was carefully put on the film surface, and its contact angle was quantified using Image J software at 25°C from its image that was taken using a digital camera (Canon, Taiwan).

### 2.11 X‐ray diffraction (XRD)

The crystalline structures were determined by XRD patterns. The following conditions were used for analysis: CuKα radiation wavelength (*λ* = 1.54 Ȧ), functioning voltage of 40 Kv and 30 mA, and 2θ diffraction angles = 3°–70°, scanning rate = 0.01 min^−1^ (Shavisi & Shahbazi, 2022).

### 2.12 Scanning electron microscope (SEM)

The microstructure of the upper surface and cryofractured cross‐section of film samples were studied by scanning electron microscope (SEM) (VP 1450; LEO‐Germany) at an accelerated voltage of 20 kV and 3000× and 5000× magnification. To make the samples conductive, the Au/Pd film, with about 30 nm thickness, was used to cover them.

### 2.13 Statistical analysis

The resulting data were analyzed by the SPSS software (IBM SPSS Statistics 26) and Microsoft Excel 2013. Data analyses were initially evaluated using a one‐way analysis of variance (ANOVA). The statistical differences between samples were assessed by multiple comparisons of means (Duncan's test, *p* ≤ .05).

## 3 RESULTS AND DISCUSSION

### 3.1 FTIR analysis

**FIGURE 1:**
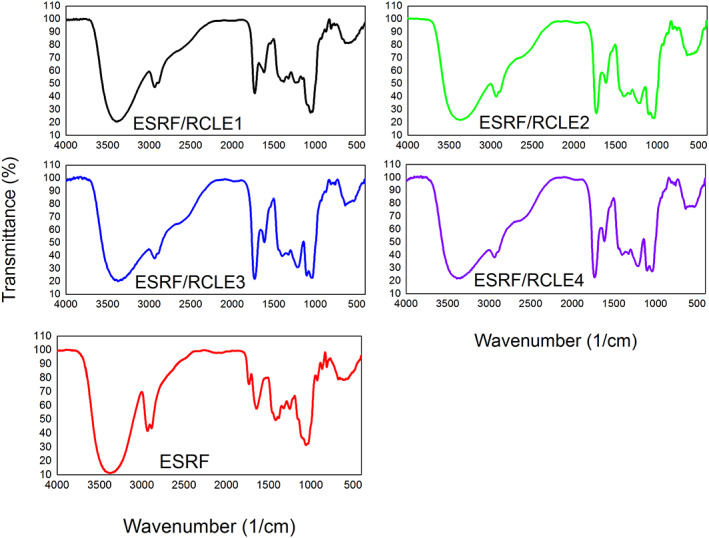
Fourier transform infrared (FTIR) spectra of *Eremurus spectabilis* root fructans (ESRF) and ESRF/*Rhus coriaria* L. extract (RCLE) films. The right numbers show the RCLE concentration.

The chemical bond interactions between the components of the film and the presence of the functional groups of polysaccharides were investigated by FTIR spectroscopy analysis as a fast and convenient method. In this technique, the structural interactions between the components of the film can be identified by the changes in the stretching vibration of the absorption bands (Thakur et al., 2016). As shown in the spectrograms of films (Figure 1), all the spectra exhibited peaks at the 600–700 cm^−1^ region, indicating the C‐H aliphatic bending. The absorption peaks in the 800–1200 cm^−1^ range suggested the presence of polysaccharides. The fingerprint region of fructans was observed at 950–1150 cm^−1^ in all spectrograms (Mozafarpour et al., 2021). According to Temkov et al. (2015), the peaks at the frequency range 1125–1162 cm^−1^ are associated with the stretching vibration of C‐O‐C. They also expressed that the absorption peak at the vicinity of 930–937 cm^−1^ (α‐D‐Glcp residue in the chain), together with both bands at 874–895 cm^−1^ (2‐ketofuranose), and 817 cm^−1^ (2‐ketose) confirm the presence of 2‐ketofuranose. As shown in Figure 1, the incorporation of RCLE increased the intensity of the absorption peaks in the 1000–1460 cm^−1^ region, corresponding to the stretching vibration of C‐OH side groups, C‐O‐C glycosidic bond, cyclic (C‐C), ether (R‐O‐R), and C‐H (CH_3_ group) bond vibrations (Pourfarzad et al., 2015). The increase in these absorption bands in composite films indicated that higher interactions occurred between the hydroxyl groups (Antoniou et al., 2014). The absorption peak at around 1730 cm^−1^ was related to the stretching vibration of the carbonyl group (Mozafarpour et al., 2021). The absorption of the C=O stretching vibration in the fructans ring appears in the spectrum at around 1640 cm^−1^ (Cordoba & Sobral, 2017; Pourfarzad et al., 2015). The enhancement of the peak intensity at 1730 cm^−1^, along with the reduction of the peak intensity at 1640 cm^−1^, with the incorporation of RCLE, indicated the presence of the carbonyl group in this extract and highlighted the tendency of carbohydrate conformation to form the intermolecular bonds (Mozafarpour et al., 2021). As seen from the spectra, these peaks also had a blue‐shift with the incorporation of RCLE, which showed the formation of intermolecular bonds in composite films (Kalaycıoglu et al., 2017). The hydrophobic and hydroxyl groups of phenolic compounds can form hydrophobic interactions and hydrogen bonds with the hydrophobic region and hydrogen acceptors of polymer molecules, respectively (Saberi et al., 2017). All the FTIR spectrograms exhibited absorption peaks at 2800, 2930, and 3300 cm^−1^ relating to the presence of the fructans. ESRF film showed very intensive absorption peaks in these regions, and the incorporation of RCLE broadened these peaks. The IR spectra showed a broad absorption band between 3000 and 3700 cm^−1^ associated with the O‐H stretching vibration of CH_2_‐OH groups from a fructofuranose unit and other components, corresponding to the free, inter‐, and intra‐molecular hydrogen bonds. The absorption peak around 2900 cm^−1^ (2800–3000 cm^−1^) indicated the symmetric and antisymmetric C‐H stretching vibration in all samples (Antoniou et al., 2014; Cordoba & Sobral, 2017; Mozafarpour et al., 2021; Temkov et al., 2015).

### 3.2 Mechanical properties

Adequate mechanical strength and extensibility are generally required for packaging films to withstand external stress and maintain integrity during application (Jridi et al., 2014). Accordingly, the mechanical properties of film samples, including the tensile strength (TS), elongation‐at‐break (EAB), and Young's modulus (YM), were investigated. The presented results in Table 1 show the incorporation of RCLE into the ESRF film had no significant effect on the EAB and TS values (*p* ˃ .05), so the brittleness of the composite films did not change significantly. However, RCLE increased the YM (*p* ˂ .05). Due to the highest YM, the ESRF/RCLE4 film exhibited the stiffest structure in microscopic images (Chambi & Grosso, 2011). According to Vilela et al., 2017, the mechanical properties of chitosan films did not change in chitosan/ellagic acid composite films. Conversely, it was stated that fructans increased the TS, EAB, and YM of whey protein isolate (WPI) films (Mozafarpour et al., 2021). However, de Moraes Crizel et al. (2018) reported that the olive residue flour, in 10%, 20%, and 30% concentration, decreased the TS, EAB, and YM of chitosan film. This fact was associated with the insoluble particles in olive flour. According to Kalaycıoglu et al. (2017), the TS and YM of chitosan films incorporated with the turmeric extract increased significantly. The TS of the distiller‐dried grains with solubles as protein (DP) film containing the tea extracts decreased, and its EAB value increased (Yang et al., 2016). Generally, polysaccharide‐based films have higher TS and lower EAB than protein‐based films (Wang et al., 2007). It was reported that the TS of sage seed gum films decreased significantly by increasing the glycerol concentration (Razavi et al., 2015). Saberi et al. (2017) reported the plasticizing effect of phenolic compounds in the starch–guar gum biocomposite edible films. This effect was attributed to the hydrogen bond formation between the phenolic compounds and polymers (Saberi et al., 2017).

**TABLE 1:** Mechanical and glass transition temperature of *Eremurus spectabilis* root fructans (ESRF) film and ESRF/*Rhus coriaria* L. extract (RCLE) composite films.

Film sample	ESRF	ESRF/RCLE1	ESRF/RCLE2	ESRF/RCLE3	ESRF/RCLE4
Tensile strength (MPa)	46.619 ± 0.725^abc^	45.781 ± 2.040^bc^	47.621 ± 1.222^ab^	49.285 ± 2.945^a^	43.975 ± 0.565^c^
Elongation‐at‐break (%)	0.956 ± 0.011^a^	1.012 ± 0.163^a^	0.997 ± 0.123^a^	0.971 ± 0.102^a^	0.904 ± 0.021^a^
Young's modulus (MPa)	31.764 ± 0.225^d^	34.356 ± 0.793^c^	33.704 ± 0.595^c^	36.734 ± 0.615^b^	37.840 ± 0.194^a^
Glass transition temperature (°C)	42.1 ± 0.60^c^	44.4 ± 1.20^b^	45.8 ± 1.50^ab^	47.1 ± 0.70^a^	48.0 ± 1.66^a^

*Note*: Different lowercase letters in the same row indicate significant differences (*p* ˂ .05). The right numbers show the RCLE concentration.

### 3.3 Glass transition temperature

Glass transition temperature (Tg) refers to the temperature at which a polymer transitions from a glassy and brittle state to a more rubbery, flexible state (Ozmen & Langrish, 2002). In food packaging, Tg is an important parameter that determines the mechanical, barrier, and sensory properties of the films. It is also an important criterion that determines the miscibility of polymers. Accordingly, the completely miscible polymers demonstrate only one Tg in DSC thermograms (Jouki et al., 2013; Suyatma et al., 2004). The incorporation of RCLE increased the Tg values significantly (Table 1). This result indicated that RCLE decreased the fructan's chain mobility by increasing the intermolecular interactions in the film matrix, which resulted in the increment of thermal stability. These results were confirmed by the SEM and mechanical properties outcomes. Namely, RCLE increased the TS and YM of the composite film. Similarly, the substitution of fructan increased the degradation and melting temperature values of WPI films (Mozafarpour et al., 2021). DSC thermograms also displayed that ESRF and RCLE can be mixed completely since only a single peak was observed in the diagrams. Conversely, it was reported that chitosan and polylactic acid cannot be used together (Suyatma et al., 2004). The type and concentration of plasticizers (Jouki et al., 2013; Mozafarpour et al., 2021; Ozmen & Langrish, 2002) and biopolymer hydrolysis (Jridi et al., 2013) also affect the Tg value of films.

The Tg values of ESRF/RCLE composite films were close to the Tg values of gum ghatti edible film (without plasticizer) but higher than the Tg values of plasticized gum ghatti films (with glycerol and sorbitol) (Ozmen & Langrish, 2002), and lower than those reported for plasticized sage seed gum films (52.2 and 179.1°C) (Razavi et al., 2015), amaranth starch granules and nanocrystals films (Condés et al., 2018), and cuttlefish skin gelatin films (50–74°C) (Jridi et al., 2013).

### 3.4 Physical properties

#### 3.4.1 Film thickness

As shown in Table 2, increasing the RCLE concentration increased the film thickness significantly over the range of 0.020–0.041 microns (*p* ˂ .05). The solid type and concentration of the film‐forming solution influence the thickness of the film (Zhang et al., 2016). The increase in the film thickness is due to the presence of the soluble material in RCLE, here the phenolic compounds, which disrupted the intermolecular bonds between the fructans chain, reorganized its structure to the more expanded structure and subsequently thickened the films (Razavi et al., 2015). Similarly, Saberi et al. (2017) reported that the natural phenolic compounds could possibly fit into the film matrix. They also related the film thickness to the density of the extract solution, which was higher than the water density.

**TABLE 2:** Physical properties and contact angle of *Eremurus spectabilis* root fructans (ESRF) film and ESRF/*Rhus coriaria* L. extract (RCLE) composite films.

Film sample	ESRF	ESRF/RCLE1	ESRF/RCLE2	ESRF/RCLE3	ESRF/RCLE4
Thickness (mm)	0.020 ± 0.005^c^	0.031 ± 0.006^b^	0.036 ± 0.005^ab^	0.042 ± 0.009^a^	0.041 ± 0.008^a^
Water content (%)	37.907 ± 1.516^a^	37.429 ± 2.539^a^	25.497 ± 2.178^b^	21.756 ± 1.578^b^	23.262 ± 3.414^b^
Solubility (%)	100.0 ± 0.00^a^	77.28 ± 1.28^b^	75.83 ± 2.61^b^	63.78 ± 1.28^c^	64.17 ± 1.67^c^
Swelling degree (%)	–	406.61 ± 37.38^a^	209.23 ± 4.09^b^	85.60 ± 2.27^c^	85.87 ± 2.65^c^
Contact angle (°)	35.629 ± 3.203^c^	42.834 ± 6.777^ab^	41.876 ± 0.949^bc^	48.993 ± 0.517^a^	41.999 ± 1.594^bc^

*Note*: Different lowercase letters in the same row indicate significant differences (*p* ˂ .05). The right numbers show the RCLE concentration.

Our results were close to the values reported for the pure chitosan, chitosan grafted with different hydroxybenzoic acids, and turmeric‐incorporated chitosan film (Kalaycıoglu et al., 2017; Liu et al., 2017), sodium alginate (1% and 1.5%) and potato starch (2%) (Wang et al., 2007), gum ghatti/glycerol films (Zhang et al., 2016), edible gelatin films (Jridi et al., 2013), but lower than those reported for WPI/fructans composite films (Mozafarpour et al., 2021), chitosan and chitosan films containing the flour and microparticles of olive pomace (de Moraes Crizel et al., 2018), protein (sodium caseinate, whey protein isolate, and gelatin) carboxymethyl cellulose (CMC) and potato starch (3%) (Wang et al., 2007), pea starch/guar gum films (Saberi et al., 2017), rice starch‐ɩ‐carrageenan films (Thakur et al., 2016), cress seed gum films (Jouki et al., 2013), gelatin, gelatin/chitosan, and gelatin/sodium caseinate films (Cordoba & Sobral, 2017).

#### 3.4.2 Water content, solubility and swelling degree

The addition of RCLE significantly decreased (*p* ˂ .05) the water content (WC) of the composite films (Table 2). This result showed that the RCLE decreased the water‐holding ability of the film matrix. This behavior could be attributed to the crosslinking interactions between fructans and RCLE (Thakur et al., 2016). Similar behavior has been reported for sorbitol in gum ghatti films. However, the glycerol behavior was completely different (Zhang et al., 2016). de Moraes Crizel et al. (2018) also reported that the addition of 30% olive pomace microparticles reduced the water content of chitosan films. This observation was attributed to the WC of the microparticles. The WC of rice starch‐ɩ‐carrageenan films was lower at the higher concentration of ɩ‐carrageenan (Thakur et al., 2016). The water content of Tara gum films was lower than those in our research (Antoniou et al., 2014).

The solubility of ESRF films was the highest (*p* ˂ .05) and decreased with increasing the concentration of RCLE. The reason for this trend could be due to the higher hydrophobicity of ESRF/RCLE films. The intermolecular interactions between the hydrophilic groups in composite films, which were confirmed with FTIR spectra, decreased their potential to interact with water molecules. Conversely, the incorporation of 30% of olive pomace increased the solubility of chitosan films (de Moraes Crizel et al., 2018).

The solubility of ESRF/RCLE films was close to the sage seed gum films (Razavi et al., 2015), higher than the solubility of gum ghatti‐based films (Zhang et al., 2016) and cress seed carbohydrate gum‐base films (Jouki et al., 2013), and lower than the solubility of cuttlefish skin gelatin films (Jridi et al., 2013). Though biodegradable films have higher water solubility (Souza et al., 2017), the low solubility increases the film resistance against high humidity and consequently improves the packaging efficiency (Chambi & Grosso, 2011). It was reported that the lipid content also affected the solubility of the edible films (Thakur et al., 2016).

Swelling power demonstrates the water absorption index (Thakur et al., 2016). RCLE concentration displayed a significant effect on the reduction of swelling degree. Because of the full solubility of the neat ESRF films, the swelling degree was not reported (Table 2). As mentioned before, due to the intermolecular interactions in ESRF/RCLE films, the availability of the hydrophilic groups to adsorb water was reduced, resulting in decreasing the WC, solubility, and swelling degree significantly. Similar results have been reported for chitosan and gelatin films incorporated with *Ziziphora clinopodioides* essential oil and grape seed extract (Shahbazi, 2017), sodium caseinate–gelatin + *Mentha spicata* L. essential oil (Eghbalian et al., 2021), and chitosan‐gum Arabic + *Rosa damascena* extract nanofiber mats (Shavisi & Shahbazi, 2022).

### 3.5 Antioxidant activity

Antioxidant packaging is a major category of active packaging that can extend the shelf life of food products. Natural antioxidants can reduce the oxidative spoilage of foods (Vilela et al., 2017). The nature and concentration of the polyphenolic compounds are generally responsible for the antioxidant activity of plant extracts (Yang et al., 2016). The main technique for inhibiting the oxidative process by antioxidants is free radical scavenging (Jridi et al., 2013). The in vitro antioxidant activity of RCLE has been reported by Nasar‐Abbas and Halkman (2004), Bursal and Köksal (2011), and Aliakbarlu et al. (2014). Kosar et al. (2007) studied the antioxidant activity and phenolic composition of RCLE. They reported the total phenolic content equal to 171.69 mg/g of Gallic acid equivalent in the methanolic extract of RCLE. Gallic acid was known as the main phenolic acid in RCLE, and its anthocyanin fraction contained cyanidin, peonidin, pelargonidin, petunidin, and delphinidin. The abundant components in the hydrolysable tannin fraction were glucosides, coumarates, and pentagalloyl glucose.

We investigated the reducing ability of the films' components by the FRAP test and free radical‐scavenging activity by the DPPH test. As shown in Table 3, the ESRF film displayed a low level of antioxidant activity. Nevertheless, as mentioned before, the *E*. *spectabilis* root has in vitro antioxidant activity. This difference is probably due to the entrance of a low concentration of root antioxidants in the ESRF film. A similar result has been reported for chitosan film (Vilela et al., 2017). Increasing the RCLE concentration increased the reducing ability and free radical‐scavenging activity of composite films. Increasing the antioxidant activity of the DP films by increasing the tea extract concentration was reported by Yang et al. (2016). The antioxidant activity of gelatin was also slightly higher than those of the resulting films (Jridi et al., 2013). Flour and microparticles of olive pomace increased the antioxidant activity of chitosan films (de Moraes Crizel et al., 2018). The presence of garlic essential oil, α‐tocopherol, and cinnamaldehyde as active compounds also increased the antioxidant activity of the gelatin, gelatin–chitosan, and gelatin–sodium caseinate films (Cordoba & Sobral, 2017). DPPH radical‐scavenging activity of ESRF is lower than the DP film, while ESRF/RCLE2, ESRF/RCLE3, and ESRF/RCLE4 have DPPH radical‐scavenging activity higher than DP film containing various types of tea extracts (Yang et al., 2016). Though having a similar trend, the DPPH radical‐scavenging activity of ESRF/RCLE2 film is higher than that reported for the chitosan/ellagic acid 5% (Vilela et al., 2017).

**TABLE 3:** Antioxidant activity of *Eremurus spectabilis* root fructans (ESRF) film and ESRF/*Rhus coriaria* L. extract (RCLE) composite films.

Film sample	ESRF	ESRF/RCLE1	ESRF/RCLE2	ESRF/RCLE3	ESRF/RCLE4
Inhibition (%) (DPPH test)	0.03 ± 0.00^d^	18.660 ± 2.470^c^	49.800 ± 5.010^b^	63.730 ± 1.250^a^	65.440 ± 4.410^a^
FRAP (μM)	0.400 ± 0.1^e^	25.400 ± 1.310^d^	58.730 ± 4.790^c^	102.070 ± 3.610^b^	117.070 ± 6.180^a^

*Note*: Different lowercase letters in the same row indicate significant differences (*p* ˂ .05). The right numbers show the RCLE concentration.

### 3.6 Water vapor permeability

In addition to solubility, water vapor permeability (WVP) is an important factor in active and intelligent packaging (Shavisi & Shahbazi, 2022). Accordingly, to increase the shelf life, water transfer should be reduced between the food product and the surrounding atmosphere (Jridi et al., 2013). The film composition, thickness, type, the extent of plasticizer, crystallinity, microstructure, and film‐making procedure affect the WVP (Liu et al., 2017; Pourfarzad et al., 2015; Thakur et al., 2016; Vilela et al., 2017). As shown in Table 4, the WVP of *E*. *spectabilis* films is significantly decreased (*p* ˂ .05) by sumac extract. The addition of 2% RCLE reduced the WVP of film samples to the moderate range of permeability values (0. 1–10 g mm/m^2^ day kPa) (Vilela et al., 2017). This fact showed again that the RCLE was well solubilized in ESRF and formed a cohesive intermolecular network that hindered the permeability of water vapor. The intermolecular interactions between the phenolic compounds of RCLE and ESRF could improve the film properties and decrease the WVP (Razavi et al., 2015). The FTIR and Tg analyses confirmed this explanation. This behavior could also be explained by the film thickness, which was directly related to the RCLE concentration. Increasing the solid density of the film matrix with increasing the RCLE concentration made the permeability of water vapor difficult. A similar explanation has recently been offered for rice starch concentration (Thakur et al., 2016). The permeability of WPI film decreased when WPI was replaced by fructans (Mozafarpour et al., 2021). In contrast, increasing the concentration of plasticizer increased the WVP of polymer films (Zhang et al., 2016). The formation of bubbles and pinholes throughout the film can also decrease its quality (Wang et al., 2007). Similarly, Yang et al. (2016) reported that black tea extract slightly decreased the WVP of the DP film. The strong interactions between ɩ‐carrageenan and rice starch reduced the WVP (Thakur et al., 2016). Grafting the different hydroxybenzoic acids onto chitosan decreased the WVP of films (Liu et al., 2017). In contrast, Vilela et al. (2017) reported that ellagic acid increased the WVP of the chitosan film.

**TABLE 4:** Water vapor transmission (WVT), permeance, and water vapor permeability (WVP) of *Eremurus spectabilis* root fructans (ESRF) film and ESRF/*Rhus coriaria* L. extract (RCLE) composite films.

Film sample	ESRF	ESRF/RCLE1	ESRF/RCLE2	ESRF/RCLE3	ESRF/RCLE4
WVT (×10^−11^ g/s.m^2^)	1.495 ± 0.168^a^	1.440 ± 0.100^a^	1.185 ± 0.0341^b^	1.083 ± 0.112^b^	1.135 ± 0.085^b^
Permeance (×10^−15^ g/Pa.s.m^2^)	4.987 ± 0.554^a^	4.790 ± 0.329^a^	3.945 ± 0.110^b^	3.612 ± 0.371^b^	3.785 ± 0.289^b^
WVP (×10^−10^ g/Pa.s.m)	2.492 ± 0.277^a^	1.542 ± 0.105^b^	1.097 ± 0.030^c^	0.859 ± 0.089^d^	0.922 ± 0.070^cd^

*Note*: Different lowercase letters in the same row indicate significant differences (*p* ˂ .05). The right numbers show the RCLE concentration.

The WVP of ESRF and ESRF/RCLE films are higher than those of the film developed by sage seed gum (Razavi et al., 2015), pure chitosan and chitosan/ellagic acid films (Vilela et al., 2017), gum ghatti films (Zhang et al., 2016), gelatin films (Jridi et al., 2013), gelatin, gelatin/chitosan, gelatin/sodium caseinate films (Cordoba & Sobral, 2017), and Tara gum films (Antoniou et al., 2014) while lower than those of the DP films containing various type of tea extract (Yang et al., 2016), chitosan films containing the flour and microparticles of olive oil pomace (de Moraes Crizel et al., 2018), and rice starch‐ ɩ‐carrageenan films (Thakur et al., 2016). ESRF/RCLE films were less permeable to water vapor than cress seed gum films, while the ESRF film permeability to water vapor was equal to those of cress seed gum (Jouki et al., 2013). The WVP of composite films (ESRF/RCLE1, ESRF/RCLE2, ESRF/RCLE3, and ESRF/RCLE4) was lower than those of chitosan grafted with different hydroxybenzoic acids films (Liu et al., 2017).

### 3.7 Contact angle

A direct relationship is generally accepted between the water contact angle and the surface hydrophobicity of the film (Cordoba & Sobral, 2017; Mozafarpour et al., 2021; Zhang et al., 2016). A hydrophobic surface has a contact angle higher than 65°, while the contact angle of a hydrophilic surface is lower than 65° (Cordoba & Sobral, 2017). Formation of the intermolecular interactions between the fructans and polyphenols, which was proved by FTIR, could change the film conformation. A similar result has been reported by increasing the fructans ratio in WPI composite films (Mozafarpour et al., 2021). According to the water contact angle, it could be concluded that the incorporation of RCLE increased the surface hydrophobicity of ESRF, but the resulting films were not completely hydrophobic and could be wetted by water. ESRF/RCLE3 film showed the highest hydrophobicity (Table 2). These results were also confirmed by the solubility and swelling degree outcomes.

The contact angle values of ESRF and ESRF/RCLE films were near to the contact angle of sage seed gum films plasticized by different concentrations of glycerol (40%–100%) (Razavi et al., 2015), cress seed gum plasticized by 35% and 50% glycerol (Jouki et al., 2013), and were lower than the contact angle of gum ghatti films (Zhang et al., 2016) and fructans/WPI composite films (Mozafarpour et al., 2021).

### 3.8 X‐ray diffraction (XRD)

**FIGURE 2:**
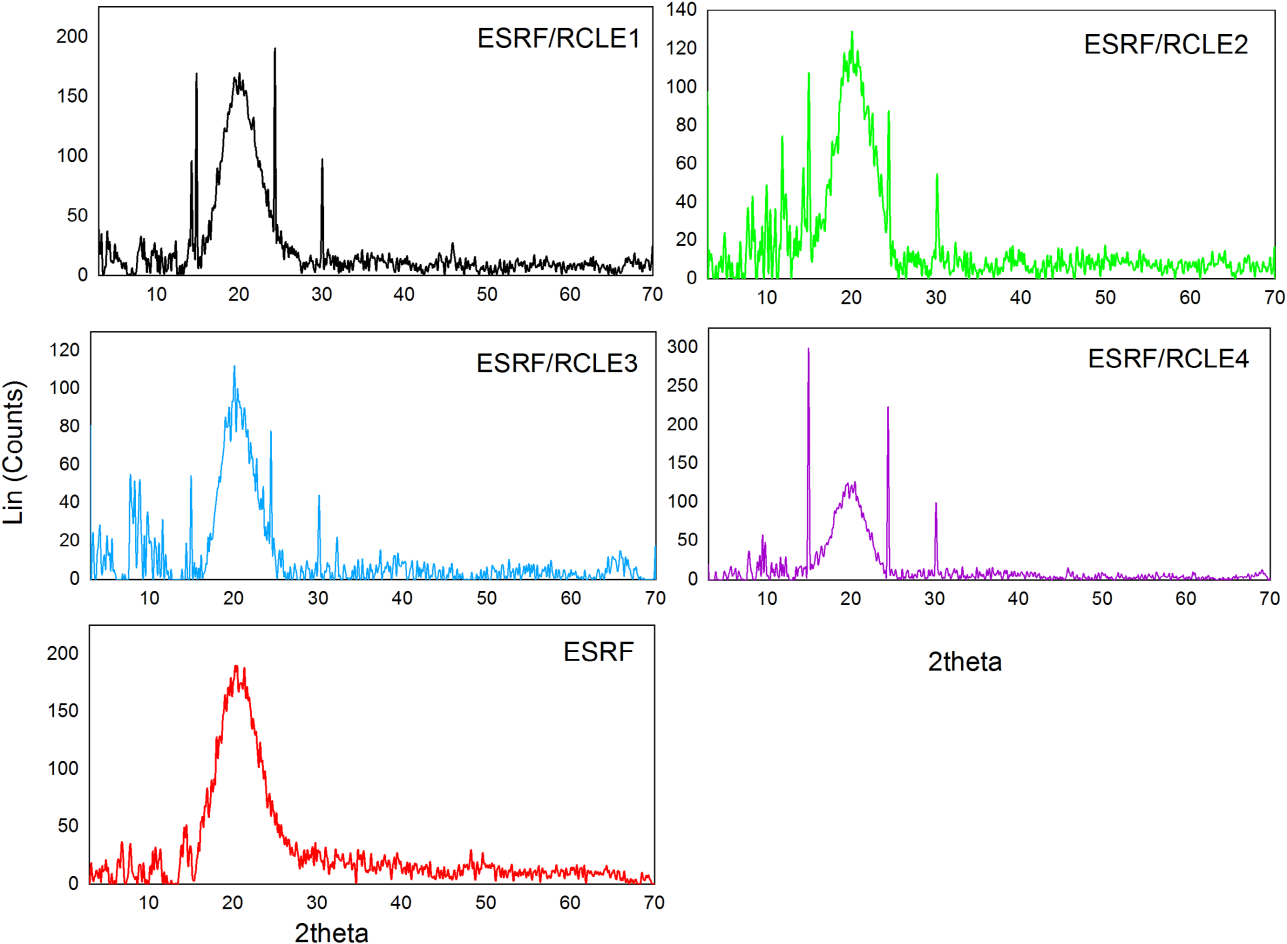
X‐ray diffraction patterns of *Eremurus spectabilis* root fructans (ESRF) and ESRF/*Rhus coriaria* L. extract (RCLE) films. The right numbers show the RCLE concentration.

In the XRD patterns, the sharp peaks present the crystalline structures, while the broad diffraction peaks demonstrate the amorphous phase. Compared with the amorphous phase, the thermodynamic stability of the crystalline structure is higher. Accordingly, the rate of dissolution of crystalline materials is lower, so their mechanical properties are different (Pourfarzad et al., 2015). According to the XRD patterns, the neat ESRF films displayed a broad peak with considerable noise at 2θ = 20.53°. Similar patterns were reported by Pourfarzad et al. (2015) and Zhang et al. (2016) for serish root fructan (spray and oven‐dried samples) and gum ghatti powder, respectively. A small peak was also observed at 2θ = 14.9°. In contrast, ESRF/RCLE films exhibited both amorphous and crystalline structures, suggesting that the formation of crystals was influenced by the presence and concentration of RCLE. An increase in the sharp reflections, an indication of crystalline structure, specifically at 2θ = 14.9°, 24.4°, and 30.1° was observed by increasing the RCLE concentration (Figure 2). As two sharp peaks at 2θ = 24.4° and 30.1° were just observed in composite film diffractograms, we concluded that they were probably associated with the RCLE crystals. It was also observed that the peak intensity at 2θ = 14.9° in composite films was higher than that in the neat ESRF films, displaying a great affinity and interaction between ESRF and RCLE (Ali et al., 2019). Once again, this fact revealed that RCLE reduced chain movement and flexibility (Zhang et al., 2016). Conversely, it was reported that plasticizers (glycerol and sorbitol) lowered the crystallinity index of gum ghatti films (Zhang et al., 2016), MgO or *Mentha spicata* essential oil (MEO) nanoparticles decreased the peak intensity of sodium caseinate‐gelatin nanofiber mats (Eghbalian et al., 2021), and *Rosa damascena* extract reduced the crystallinity index of chitosan–gum Arabic nanofiber mats (Shavisi & Shahbazi, 2022). Compared with the neat ESRF films, the higher crystallinity indicated the greater strength of composite films. This result was in correspondence with Young's modulus of samples. Similar results were reported for the addition of the cellulose nanocrystals into the seaweed nanocomposite films (Doh et al., 2020) and starch‐based films reinforced by pomegranate peel (Ali et al., 2019).

### 3.9 Scanning electron microscope (SEM)

**FIGURE 3:**
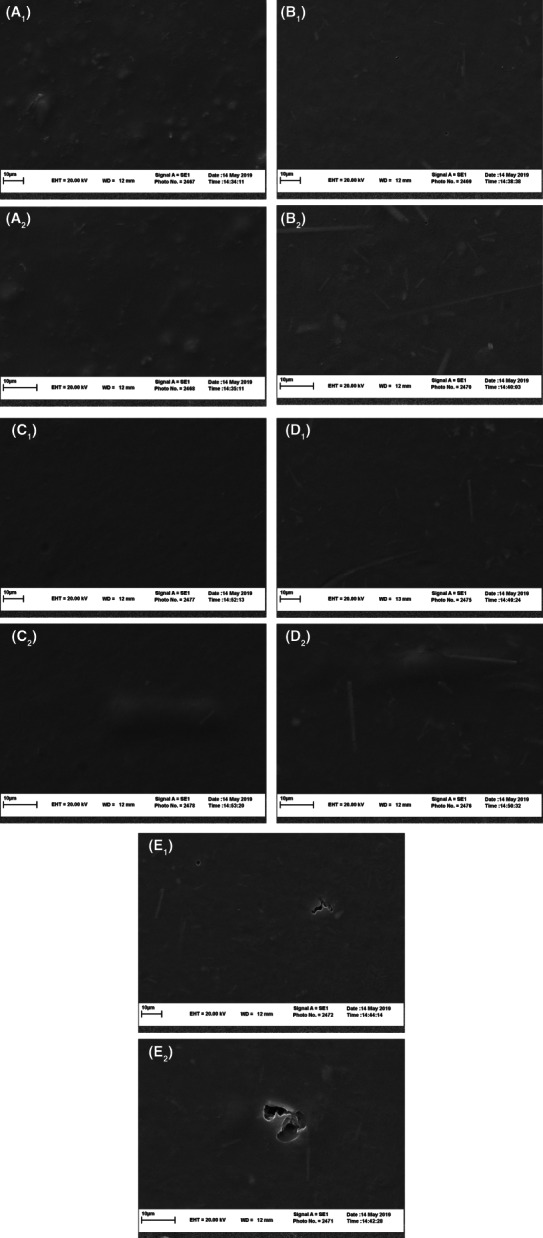
The microstructure of upper surface of *Eremurus spectabilis* Root fructans (ESRF) film (A_1_, A_2_), and ESRF/*Rhus coriaria* L. extract (RCLE) films at 1% (B_1_, B_2_) 2% (C_1_, C_2_), 3% (D_1_, D_2_) 4% (E_1_, E_2_) of RCLE concentrations and 3000× (A_1_, B_1_, C_1_, D_1_, and E_1_) and 5000× (A_2_, B_2_, C_2_, D_2_, and E_2_) magnifications.

**FIGURE 4:**
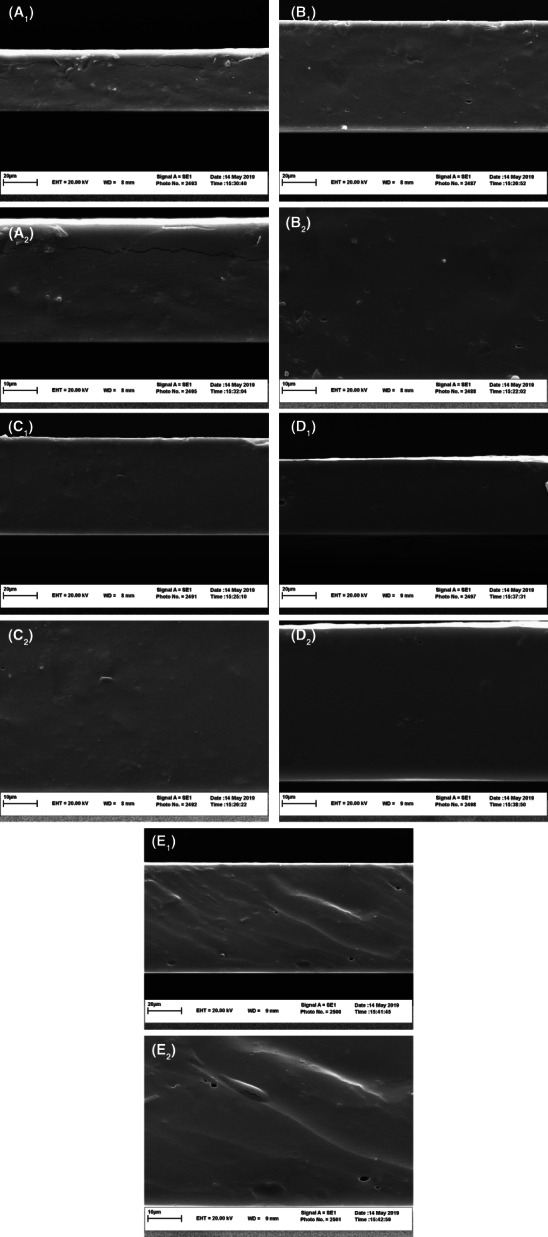
The microstructure of cryo‐fractured cross‐section of *Eremurus spectabilis* Root fructans (ESRF) film (A_1_, A_2_) and ESRF/*Rhus coriaria* L. extract (RCLE) films at 1% (B_1_, B_2_), 2% (C_1_, C_2_), 3% (D_1_, D_2_), 4% (E_1_, E_2_) of RCLE and 3000× (A_1_, B_1_, C_1_, D_1_, and E_1_) and 5000× (A_2_, B_2_, C_2_, D_2_, and E_2_) magnifications.

The effect of the RCLE addition to the ESRF film was visualized using the scanning electron microscope (SEM). It presented a relationship between the microstructural properties and the physical characteristics of films (de Moraes Crizel et al., 2018; Mozafarpour et al., 2021; Zhang et al., 2016). As can be seen in Figures 3 and 4, the ESRF film exhibited a rough surface and obvious cracks on the cross‐section. However, the incorporation of the RCLE resulted in a uniform and more compact surface without the cross‐section cracks. The formation of intermolecular bonds in composite films facilitates the miscibility of RCLE and ESRF. This phenomenon caused lower WVP values in composite films than in pure fructan film with a rough surface. The more compact and denser surface and cross‐section were visualized with a 3% RCLE addition. The film cross‐section at 4% RCLE concentration was rough and bumpy, which was probably due to an increase in the viscosity of the film‐forming solution. Therefore, the surface properties of ESRF film can be improved by the RCLE.

## 4 CONCLUSION

Different concentrations of *Rhus coriaria* L. extract (RCLE) were used to improve the properties of the *E*. *spectabilis* root fructans (ESRF) film. The FTIR spectra proved the formation of intermolecular bonds in composite films. The incorporation of RCLE into the ESRF film had no significant effect on its brittleness, but it increased the film stiffness, thermal stability, and crystallinity. Increasing the RCLE concentration increased the film thickness, the reducing ability, and free radical‐scavenging activity. It also decreased the water content, solubility, and swelling degree of composite films significantly. The intermolecular interactions between the phenolic compound of RCLE and ESRF improved the microstructural properties and decreased the WVP of the films. RCLE also increased the surface hydrophobicity, but the resulting films were not completely hydrophobic and could be wetted by water. It can be concluded that the ESRF/RCLE active films at the 3% concentration of *Rhus coriaria* L. extract are suggested as a good active packaging material with suitable properties.

## AUTHOR CONTRIBUTIONS

**Somayeh Pakseresht:** Conceptualization (equal); data curation (equal); formal analysis (equal); investigation (equal); methodology (equal); software (equal); writing – original draft (equal); writing – review and editing (equal). **Jouhaina Hadree:** Conceptualization (equal); data curation (equal); formal analysis (equal); investigation (equal); methodology (equal); writing – original draft (equal); writing – review and editing (equal). **Nasser Sedaghat:** Funding acquisition (equal); methodology (equal); project administration (equal); supervision (equal); writing – review and editing (equal).

## CONFLICT OF INTEREST STATEMENT

The authors declare that they do not have any conflict of interest.

## ETHICAL APPROVAL

This study does not involve any human or animal tasting.

## Data Availability

All the necessary data generated and/or analyzed during the current study are included in this published article and its additional information, if needed, is available from the corresponding author on reasonable request.
